# *Plasmodium falciparum* infection and clinical indicators in relation to net coverage in central Côte d’Ivoire

**DOI:** 10.1186/1756-3305-7-306

**Published:** 2014-07-03

**Authors:** Allassane F Ouattara, Mamadou Dagnogo, Piero L Olliaro, Giovanna Raso, Marcel Tanner, Jürg Utzinger, Benjamin G Koudou

**Affiliations:** 1 Département Environnement et Santé, Centre Suisse de Recherches Scientifiques en Côte d’Ivoire, 01 BP 1303, Abidjan 01, Côte d’Ivoire; 2 Laboratoire de Cytologie et de Biologie Animale, Unité de Formation et de Recherche Sciences de la Nature, Université Nangui Abrogoua, 02 BP 801, Abidjan 02, Côte d’Ivoire; 3 Department of Epidemiology and Public Health, Swiss Tropical and Public Health Institute, P.O. Box, CH-4002 Basel, Switzerland; 4 University of Basel, P.O. Box, CH-4003 Basel, Switzerland; 5 UNICEF/UNDP/World Bank/WHO Special Programme for Research and Training in Tropical Diseases (TDR), 20 avenue Appia, CH-1211 Geneva 27, Switzerland; 6 Centre for Neglected Tropical Diseases, Pembroke Place, Liverpool L3 5QA, UK

**Keywords:** *Plasmodium falciparum*, Fever, Long-lasting insecticidal net, Prevalence, Microscopy, Rapid diagnostic test, Côte d’Ivoire

## Abstract

**Background:**

Sleeping under a net, particularly a long-lasting insecticidal net (LLIN), is associated with reduced malaria morbidity and mortality, but requires high coverage and adherence. In this study, parasitologically confirmed *Plasmodium falciparum* infection and a clinical indicator (i.e. fever) were measured among children in three villages of central Côte d’Ivoire (Bozi, N’Dakonankro and Yoho) and associations with net coverage explored. In Bozi and Yoho, LLINs were provided by the national malaria control programme, prior to the study and an additional catch-up coverage was carried out in Bozi. In N’Dakonankro, no net intervention was conducted.

**Methods:**

Three cross-sectional surveys were carried out; two in the dry season (February 2010 and November 2011) and one in the rainy season (May 2012). Among 897 children aged <15 years, *P. falciparum* infection was determined by microscopy and a rapid diagnostic test (RDT). Fever was defined as an axillary temperature ≥37.5°C. A questionnaire was administered to obtain demographic data and net usage.

**Results:**

The proportion of children infected with *P. falciparum* according to microscopy in the third survey was 74%, 81% and 82% in Yoho, N’Dakonankro and Bozi, respectively. Meanwhile, 46% of the children in N’Dakonankro, 44% in Bozi and 33% in Yoho slept under a net. The risk of *P. falciparum* infection did not differ between net-sleepers and non-net-sleepers. Fewer children had parasitaemia ≥1,000 parasites/μl of blood in Bozi in the third compared to the first survey. Fever was poorly correlated with *P. falciparum* infection. The risk of *P. falciparum* infection did not depend on the village of residence, presence of fever or sleeping under LLIN the night before the survey. Conversely, it was higher in the rainy season and among older children.

**Conclusions:**

In an area where *P. falciparum* is highly prevalent, the use of nets was associated with significantly lower levels of parasitaemia. The apparent lack of effect on *P. falciparum* infection and fever might be explained by the relatively low net coverage in Bozi and Yoho and the relatively short period (<2 years) during which the impact of nets was measured.

## Background

Controlling malaria remains a formidable public health challenge, particularly in sub-Saharan Africa, where 80% of all cases and 90% of all malaria-attributable deaths occur, mainly among children below the age of 5 years [1]. Malaria control efforts have been stepped up worldwide, facilitated by the availability and implementation of tools with a proven track record to prevent (e.g. insecticide-treated nets (ITNs) and, more recently, long-lasting insecticidal nets (LLINs)), accurately diagnose (rapid diagnostic tests (RDTs)) and treat malaria (artemisinin-based combination therapy (ACT)). The use of ITNs and LLINs proved effective in reducing vector density and malaria transmission, infection prevalence and febrile episodes [2-4], but it requires adequate use and high coverage [5]. However, the effective protection of ITNs against mosquito bites has been shown to decay over time both physically (hole formation) and chemically (insecticide loss) [6,7]; the rate of this decay in the efficacy of ITNs tends to accelerate after 2–3 years with a high proportion of holes [8]. RDTs are now increasingly reliable [9] and available [10], although their performance in the field, the prescriber’s reliance on RDT results, and the patients’ perception about RDTs still vary from one setting to another [10-12]. Regardless of the overall efficacy, these tools will not achieve the set goals of malaria control and elimination, unless adequately distributed and used at a high coverage. Indeed, high coverage of the populations at risk for malaria by ITNs, LLINs, RDTs and ACT is essential to reduce the malaria burden and to achieve malaria elimination in selected areas.

In countries like Côte d’Ivoire, the challenge now is to roll out interventions at a level sufficient to gain and maintain effective malaria control. With regard to LLINs, they were distributed from 2006 to 2007 to children aged <11 months, along with routine immunization, and to pregnant women at antenatal clinics in 37 health districts. In 2008, LLINs distributions took place in 19 health districts with financial support of the Global Fund to Fight AIDS, Tuberculosis and Malaria to children aged <11 months at health centres, and to all pregnant women attending antenatal clinics. In the same year, an additional 18 health districts benefitted from LLINs during measles vaccination campaigns of 5-year-old children and vitamin A supplementation. In 2009, the national coverage of LLINs was estimated at 24.4% and the usage rate at 16.3%. The coverage and usage rate of LLINs of pregnant women was 19.8% and 13.9%, respectively, whilst the respective percentages for children under the age of 5 years were 30.2% and 28.1%. In Bouaflé health district in central Côte d’Ivoire, LLINs coverage was 47.4% and usage rate 20.2% [13]. The recent annual report from the ‘Programme National de Lutte contre le Paludisme’ (PNLP) confirmed a reluctance to use nets in this area [14].

This study was part of a broader project aimed at measuring the impact of nets on malaria transmission, as determined by entomological inoculation rate (EIR), and different parasitological and clinical indicators, such as prevalence, parasitaemia and morbidity due to *Plasmodium* infection. The key findings pertaining to malaria transmission have been presented elsewhere [15]. In brief, high coverage and sensitization of households to use nets resulted in highly significant reductions of the EIR. The purpose of the current study was to determine possible associations between two key malaria indicators (i.e. *P. falciparum* infection and fever) and net coverage, during the dry and rainy seasons in rural settings of central Côte d’Ivoire with different levels of malaria transmission and vector control interventions. Two villages (Bozi and Yoho) are located in the health district of Bouaflé and received LLINs by the PNLP. Additionally, in Bozi, LLINs were distributed in a catch-up intervention to all households inhabited by children aged <15 years and pregnant women. In the third village (N’Dakonankro), no specific net interventions were carried out. As RDTs had been introduced in Côte d’Ivoire and were brought to scale, we also wanted to compare the performance of RDT *versus* microscopy for the diagnosis of *P. falciparum* infection.

## Methods

### Ethical considerations

The study protocol was reviewed by the institutional review board of the Centre Suisse de Recherches Scientifiques en Côte d’Ivoire (CSRS; Abidjan, Côte d’Ivoire). Ethical approval was granted by the national ethics committee of Côte d’Ivoire (reference no. 02-2011/MSLS/CNER-P). Oral informed consent was obtained from parents/guardians of participating children and heads of households, whilst children provided oral assent. Oral rather than written informed consent was obtained because of high illiteracy rates in rural Côte d’Ivoire. The purpose and procedures of the study were explained to all participating households in the local language using lay terms. It was emphasised that participation is voluntary and people could withdraw anytime without further obligation. Children with a positive RDT and axillary temperature ≥37.5°C were given an ACT (i.e. artemether-lumefantrine) free of charge.

### Study area

**Figure 1:**
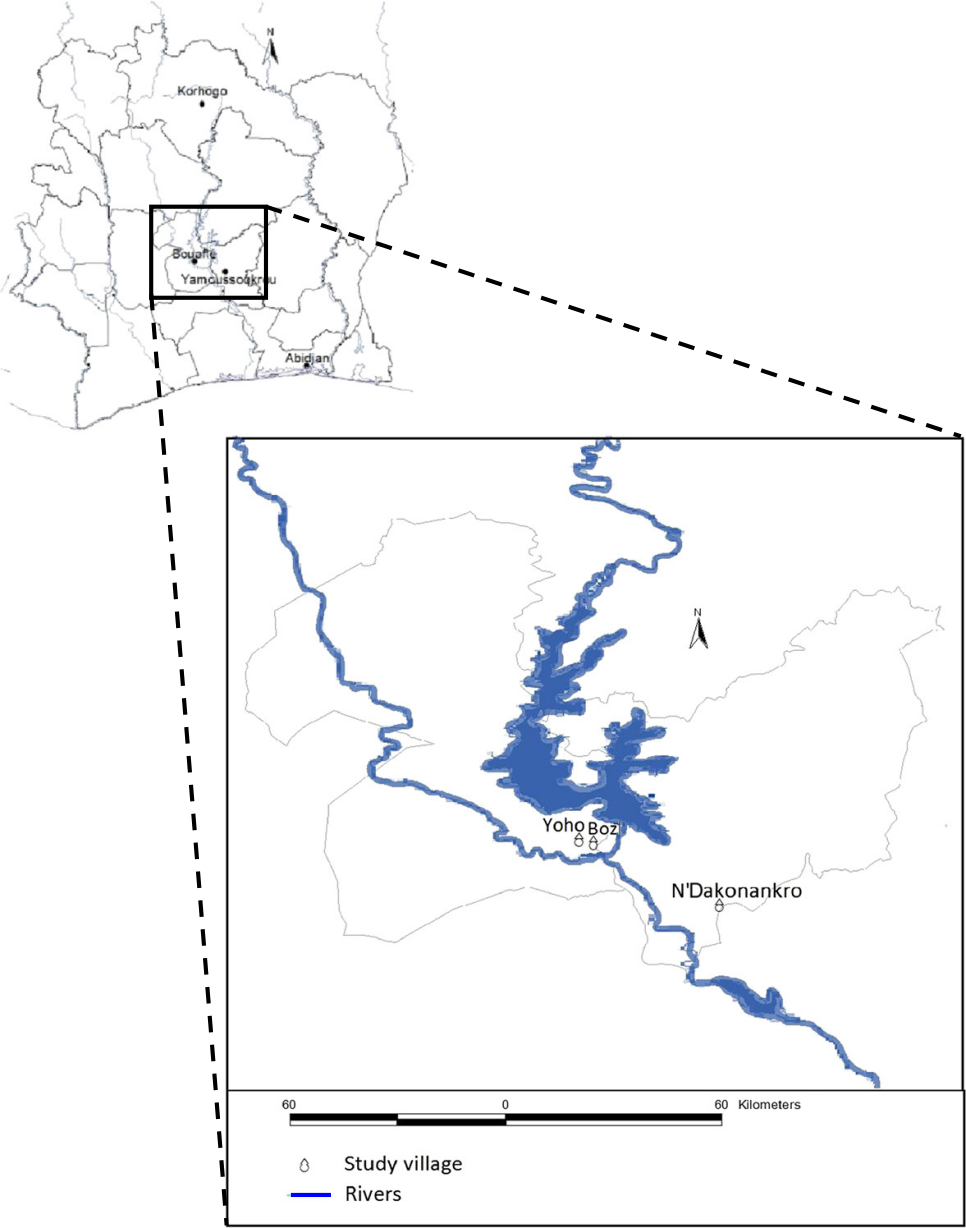
The three study villages in central Côte d’Ivoire.

The study was carried out between July 2009 and May 2012 in three villages of central Côte d’Ivoire; Bozi (geographical coordinates 6°55.151’ N latitude, 5°32.080’ W longitude; 2,354 inhabitants); N’Dakonankro (6°45.560’ N, 5°13.195’ W; 827 inhabitants) and Yoho (6°55.364’ N, 5°34.569’ W, 2,624 inhabitants) [15]. N’Dakonankro is located near Yamoussoukro, the capital of Côte d’Ivoire; whilst Bozi and Yoho are situated in the department of Bouaflé, separated by a distance of 5 km (Figure 1). The monthly temperatures range between 26°C and 29°C and the mean humidity is between 70% and 80% in the rainy season. The annual precipitation during 2009–2011 was 1,181 mm in N’Dakonankro and 1,236 mm in Bozi and Yoho with peak rainfalls observed between mid-March and mid-July and September/October (SODEXAM database, 2012). Houses in N’Dakonankro were built in cement and have electricity, while in Bozi and Yoho houses were made either of cement or mud with a straw roof. Irrigated rice farming in close proximity to human settlements is practiced in all three villages.

In central Côte d’Ivoire *Plasmodium* parasite prevalence ranges between 40% and 86% [16]. There is perennial and intense transmission, with a peak during the rainy season. The EIR in irrigated rice farming areas has been estimated at 295–572 infectious bites per person per year [17].

### Free LLIN distribution in Bozi and households survey

**Figure 2:**
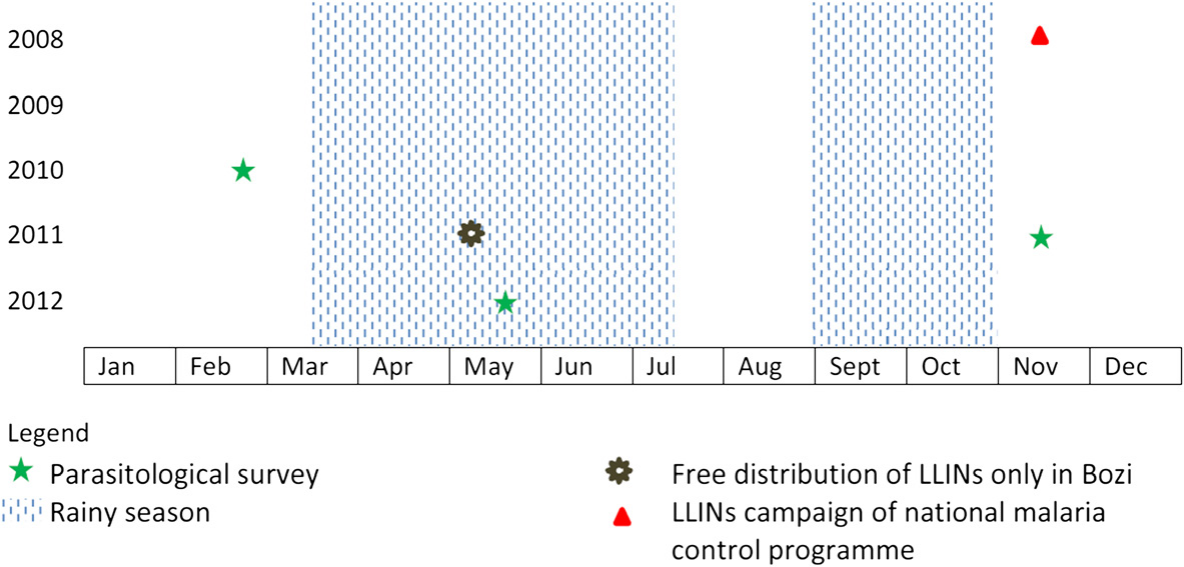
Study design and timing of parasitological survey and free distribution of LLINs from 2009 to 2012 in three rural communities of central Côte d’Ivoire.

A LLIN campaign was implemented in November 2008 by the PNLP, and both Bozi and Yoho benefited from this intervention (Figure 2). In July 2009, a survey revealed low LLIN coverage rates (35.2% in Bozi, 10.2% in Yoho) [18]. N’Dakonankro did not benefit from this intervention because, at the time, the free distribution of LLINs by the PNLP was restricted to villages with very high prevalence rates of malaria. Moreover, the district of Yamoussoukro (where N’Dakonankro is located) intended to run an indoor residual spraying (IRS) campaign but it never took place because of the sociopolitical turmoils and armed conflict [19]. Coverage of other types of nets (mainly ITNs) in N’Dakonankro was low (7.1%).

In May 2011, with the help of community health workers (CHWs), an additional 150 LLINs were distributed to households in Bozi inhabited by children aged <15 years and pregnant women. Households were coded and clearly marked after receipt of a LLIN. Household heads were instructed how to use and wash LLINs.

### Study design and timing

Figure 2 shows the design of the study. A baseline cross-sectional survey was conducted among children aged below 15 years in February 2010 (end of the long dry season). In May 2011, a catch-up intervention of LLINs was carried out in Bozi. Data were collected at months 6 and 12 after LLIN distribution in November 2011 (right after the end of the short rainy season) and May 2012 (during the main rainy season).

### Blood sample collection

**Figure 3:**
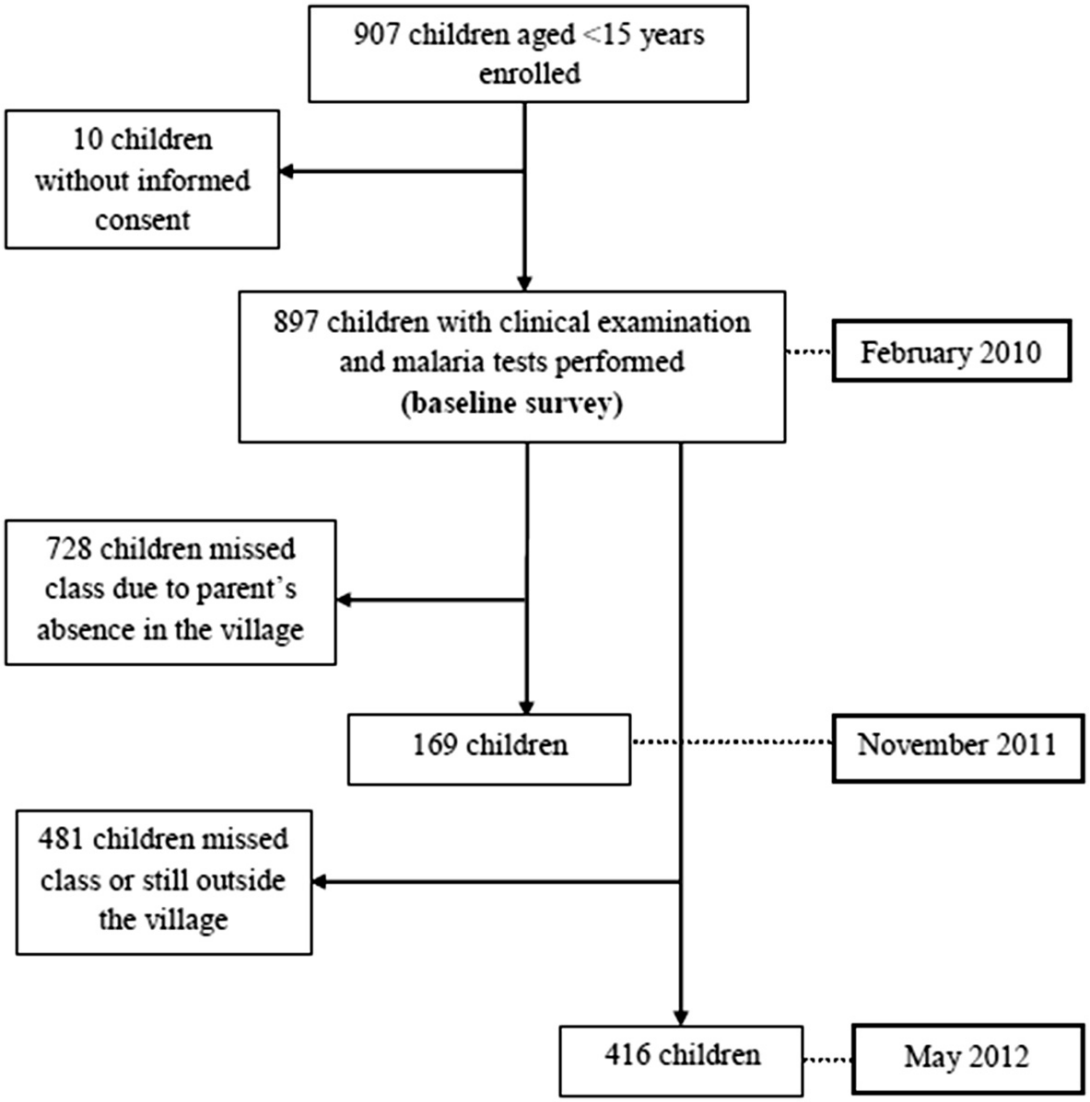
Flow chart detailing the study participation and compliance with finger-prick blood sampling in three villages of central Côte d’Ivoire.

In February 2010, a baseline cross-sectional survey was carried out in the three villages. All children aged between 7 and 15 years who attended grades 1–4 in the primary schools were invited for a finger-prick blood sample. Furthermore, mothers and caregivers of under 7-year-old children were invited to bring their children to a designated community location where finger-prick blood samples were taken. Net use was reported by schoolchildren themselves and the team considered these nets as LLIN because they had been offered by the PNLP. A total of 897 children participated, corresponding to 98.9% of those invited (n = 907). In November 2011, 6 months after free LLIN catch-up in Bozi, a second survey was carried out, including 169 children. One year later, in May 2012, a third survey enrolled 416 children. Many children were absent during the second and third survey (Figure 3).

### Blood films and RDT preparation

Thick and thin blood films were prepared on microscope slides and an HRP-2 based RDT (ICT ML01 malaria *Pf* test; ICT Diagnostics, Cape Town, South Africa) were performed at the same time. The slides were air-dried and then transferred to a nearby laboratory where they were stained with Giemsa for 45 min. Slides were examined under a microscope at high magnification by the same experienced technician throughout the study. *Plasmodium* density was estimated by counting the number of parasites per 200 leucocytes. Whenever fewer than 10 parasites were recorded, reading was continued for a total of 500 leucocytes. Parasite count was converted to parasites/μl of blood, assuming a standard count of 8,000 leucocytes/μl of blood. For quality control, 10% of the slides were randomly selected and re-examined by a senior technician.

### Statistical analysis

Statistical analyses were performed using STATA version 10 (Stata Corporation; College Station, TX, USA). Clinical malaria was defined as children with either a positive RDT or a positive Giemsa-stained microscope slide (or both) plus axillary temperature ≥37.5°C. Prevalence was the number of positive children divided by the total number of children examined. We used the results from the thick and thin blood film examinations for estimating the prevalence and the relationship with net coverage.

Sensitivity, specificity and predictive values of the ICT ML01 malaria *Pf* test were calculated, considering microscopy as the diagnostic reference standard. The kappa statistic was used to assess the concordance between ICT ML01 malaria *Pf* test and microscopy. Univariate logistic regression was employed to assess the effect of one variable on risk of *P. falciparum* infection. A multivariable model was used to assess the overall effect of the aforementioned variables. Chi-square (χ^2^) or Fisher’s exact test, as appropriate, was used to compare groups.

## Results

### Main characteristic of the study population

Overall, there were 1,482 contacts, equally distributed across the three villages on the three occasions (483 in N’Dakonankro, 499 in Yoho and 500 in Bozi); 61% (95% confidence interval (CI) 58-63%) occurred on the first survey, 11% (95% CI 10-13%) on the second (dry season) and 28% (95% CI 26-30%) on the third (rainy season). The mean age of surveyed children ranged from 6.7 (95% CI 6.4-6.9) to 8.7 (95% CI 8.1-9.4) years at the first survey, and from 10.7 (95% CI 10.5-10.8) to 12.2 (95% CI 11.7-12.6) years at the third survey (Table 1). Few children were <5 years old at the first survey (N’Dakonankro, n = 52, 17.3%, 95% CI 13.2-22.0%; Yoho, n = 35, 12.2%, 95% CI 8.6-16.4%; and Bozi, n = 64, 20.9%, 95% CI 16.5-25.9%) and none at the third survey. The age of subjects differed between the three villages (p < 0.001) and increased over time within the same site (p < 0.001).

**Table 1:** Characteristics of study population

**Characteristic**	**N’Dakonankro**	**Yoho**	**Bozi**	**P value**
***February 2010***				
No. of subjects	301	290	306	
No. of males:females (%)	151:150 (50:50)	150:140 (52:48)	146:160 (48:52)	0.613
Mean age in years (standard deviation)	6.7 (2.3)	7.6 (2.6)	8.7 (5.7)	<0.001^†^
No. of children who slept under an ITN or LLIN the night before the interview (%, 95% CI)	75 (24.9, 20.1-30.2)	44 (15.2, 11.2-19.8)	66 (21.6, 17.1-26.6)	0.012^†^
No. of febrile cases (%, 95% CI)	145 (48.2, 42.4-54.0)	123 (42.4, 36.7-48.3)	104 (34.0, 28.7-39.6)	0.002^†^
No. of slide positive (%, 95% CI)	167 (55.5, 49.7-61.2)	187 (64.5, 58.7-70.0)	140 (45.8, 40.1-51.6)	<0.001^†^
No. of RDT positive (%, 95% CI)	186 (61.8, 56.0-67.3)	190 (65.5, 59.7-71.0)	174 (56.9, 51.1-62.5)	0.093
Geometric mean of parasitaemia, expressed in parasites/μl of blood (95% CI)	37.5 (26.0-54.1)	79.9 (54.7-116.6)	18.5 (12.8-26.7)	
Sensitivity; specificity; PPV; NPV (%, 95% CI)	79.6 (75.7-83.0); 61.0 (56.1-65.8); 71.5 (67.5-75.2); 70.9 (65.8-75.6)			
***November 2011***				
No. of subjects	45	56	68	
No. of males:females (%)	25:20 (56:44)	42:14 (75:25)	35:33 (52:48)	0.021^†^
Mean age in years (standard deviation)	9.3 (1.7)	9.4 (1.8)	10.3 (3.2)	0.010
No. of children who slept under an ITN or LLIN the night before the interview (%, 95% CI)	21 (46.7, 31.7-62.1)	28 (50.0, 36.3-63.7)	21 (30.9, 20.2-43.3)	0.070
No. of febrile cases (%, 95% CI)	2 (4.4, 0.5-15.1)	6 (10.7, 4.0-21.9)	4 (5.9, 1.6-14.4)	0.485
No. of slide positive (%, 95% CI)	35 (77.8, 63.0-88.8)	40 (71.4, 57.8-82.7)	59 (86.8, 76.4-93.8)	0.156
No. of RDT positive (%, 95% CI)	29 (64.4, 48.8-78.1)	38 (67.9, 54.0-79.8)	47 (69.1, 56.8-79.8)	0.909
Geometric mean of parasitaemia, expressed in parasites/μl of blood (95% CI)	118.9 (50.1-282.2)	78.9 (35.9-173.4)	136.0 (77.3-239.0)	
Sensitivity; specificity; PPV; NPV (%, 95% CI)	80.6 (72.9-86.9); 83.3 (67.2-93.6); 94.7 (88.9-98.0); 53.6 (39.7-67.0)			
***May 2012***				
No. of subjects	137	153	126	
No. of males:females (%)	71:66 (52:48)	77:76 (50:50)	73:53 (58:42)	0.418
Mean age in years (standard deviation)	10.7 (0.9)	11.3 (2.0)	12.2 (2.5)	<0.001^†^
No. of children who slept under an ITN or LLIN the night before the interview (%, 95% CI)	63 (46.0, 37.4-54.7)	50 (32.7, 25.3-40.7)	55 (43.7, 34.8-52.8)	0.047^†^
No. of febrile cases (%, 95% CI)	4 (2.9, 0.8-7.3)	3 (2.0, 0.4-5.6)	5 (4.0, 1.3-9.0)	0.621
No. of slide positive (%, 95% CI)	111 (81.0, 73.4-87.2)	113 (73.9, 66.1-80.6)	102 (81.6, 73.0-87.4)	0.203
No. of RDT positive (%, 95% CI)	96 (70.1, 61.7-77.6)	108 (70.6, 62.7-77.7)	79 (63.2, 53.6-71.1)	0.356
Geometric mean of parasitaemia, expressed in parasites/μl of blood (95% CI)	158.4 (104.1-241.1)	68.7 (45.5-103.7)	125.9 (81.3-194.8)	
Sensitivity; specificity; PPV; NPV (%, 95% CI)	76.1 (71.1-80.6); 60.7 (49.7-70.9); 87.6 (83.2-91.2); 40.9 (32.4-49.8)			

† Significant.

We planned for 10% of the 1,482 thick blood films to be read by two independent senior microscopists for quality control purposes; of the 148 paired readings (90, 17 and 41 at the first, second and third survey, respectively), 144 matched (89, 16 and 39, respectively), for a between-reader agreement of 98.8% (95% CI 94.0-100%), 94.1% (95% CI 71.3-99.9%) and 95.1% (95% CI 83.5-99.4%) for the three surveys, respectively. Discrepancies occurred for slides with low parasitaemia.

### *Plasmodium falciparum* infection risk

Based on microscopy, the risk of being infected with *P. falciparum* differed across the three villages at the end of the dry season (first survey). A higher prevalence was found in Yoho (64.5%, 95% CI 58.7-70.0%) compared to N’Dakonankro (55.5%, 95% CI 49.7-61.2%) and Bozi (45.8%, 95% CI 40.1-51.6%) (both p < 0.001) (Table 1). The risk differed significantly over time (p < 0.001) and between sites (p = 0.031). The 416 subjects who underwent a parasitological examination on both the first and the third survey, 57.0% (95% CI 48.2-65.4%), 63.4% (95% CI 55.2-71.0%) and 52.4% (95% CI 43.1-61.3%) were found to be positive at the first survey in N’Dakonankro, Yoho and Bozi; these proportions increased at the third survey. A significant difference was observed in individual shifts in status (positive or negative) only in Bozi, with more children being observed slide-positive (Table 2) (p < 0.05).

**Table 2:** Shifts in status (positive or negative slides) at three villages of central Côte d’Ivoire

		**First survey**			**χ**^**2**^
		**Negative (%)**	**Positive (%)**	**Total (%)**	
**Third survey**	N’Dakonankro				
	Negative (%)	15 (10.9)	11 (8.0)	26 (19.0)	
	Positive (%)	44 (32.1)	67 (49.0)	111 (81.0)	
	All (%)	59 (43.0)	78 (57.0)	137 (100)	0.094
	Yoho				
	Negative (%)	16 (10.5)	24 (15.7)	40 (26.1)	
	Positive (%)	40 (26.1)	73 (47.7)	113 (73.9)	
	All (%)	56 (36.6)	97 (63.4)	153 (100)	0.604
	Bozi				
	Negative (%)	17 (13.5)	8 (6.4)	25 (19.8)	
	Positive (%)	43 (34.1)	58 (46.0)	101 (80.2)	
	All (%)	60 (47.6)	66 (52.4)	126 (100)	0.027†

† Significant.

### Risk of fever

The risk of fever was highest at the first survey, and significantly lower in Bozi (34.0, 95% CI 28.7-39.6%) than in the other two villages (p = 0.002) (Table 1); it dropped significantly at the second and third survey in the three villages (no between-village difference). For the 416 children who were seen on both the first and third surveys, only 2.9% (95% CI 0.8-7.3%) had fever on the latter occasion. No significant difference was observed in fever risk (p > 0.05) across villages.

### Net usage

The percentage of children who reportedly had slept under a net the night before the first survey was 24.9% (95% CI 20.1-30.2%) in N’Dakonankro (mainly ITNs), 15.2% (95% CI 11.2-19.8%) in Yoho (mainly LLINs) and 21.6% (95% CI 17.1-26.6%) in Bozi (mainly LLINs) (p = 0.012; difference accounted for by Yoho) (Table 1). At the third survey, a borderline difference was observed between villages (p = 0.047; difference accounted for by Yoho). The net coverage was different between villages (p = 0.010) and over time (p < 0.001).

For the 416 children who were seen on the first and the third survey, 23.4% (95% CI 16.6-31.1%), 24.2% (95% CI 17.6-31.8%) and 26.9% (95% CI 19.5-35.6%) reported to have slept under a net the night before the interview in N’Dakonankro, Yoho and Bozi, respectively. A significant difference was observed between surveys only in Yoho (p = 0.031).

### Relationship between net use and *P. falciparum* infection

**Figure 4:**
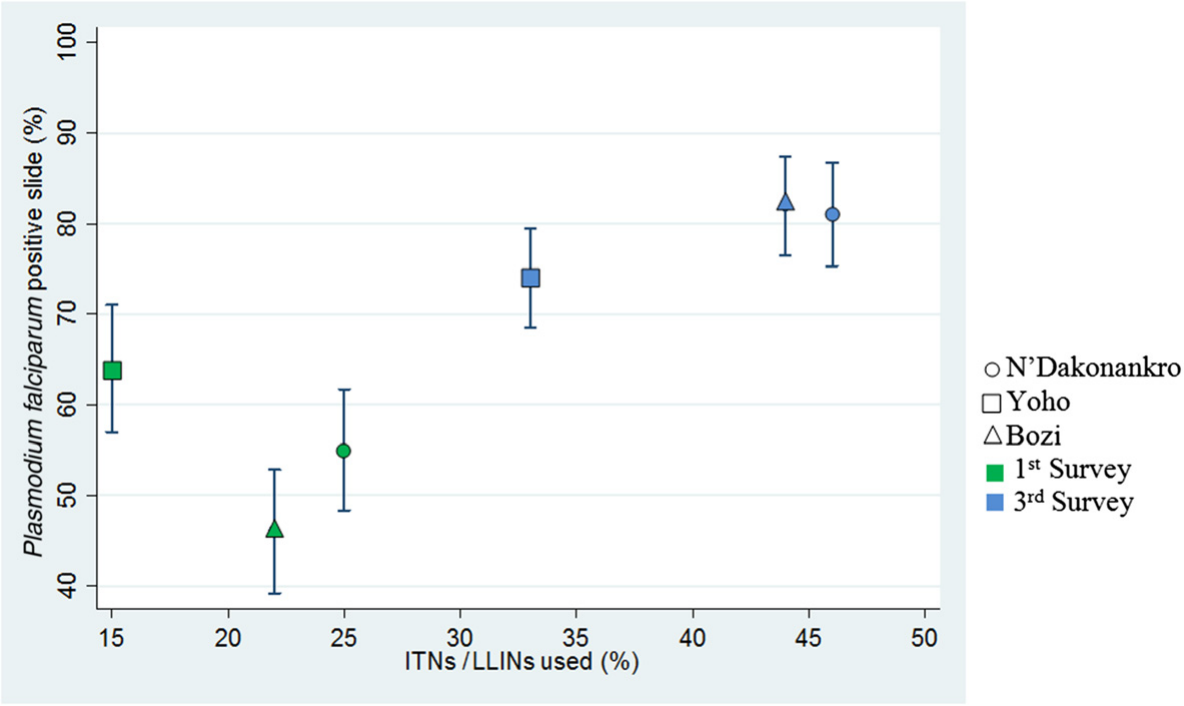
Relationship between percentage of positive thick and thin blood films and LLINs used among children aged <15 years in three villages of central Côte d’Ivoire.

Figure 4 shows the relationship between the rate of net usage and *P. falciparum* infection (determined by microscopy) during the first and the third survey in the three study villages. At the third survey in N’Dakonankro, 81% of children were infected and 46% slept under a net (mainly ITNs), while in Yoho, the respective figures were 74% and 33% (mainly LLINs) and in Bozi, 82% and 44% (mainly LLINs) (Figure 4). The prevalence of infection among children who slept under a net the night before the interview did not differ across the sites at any time (p > 0.05). In addition, the risk of *P. falciparum* infection did not differ between net-sleepers and non-net-sleepers (odds ratio (OR)=1.17, 95% CI 0.92-1.48).

### Relationship between net use and fevers

Children who had fever on the day of survey and had slept under a net were 20%, 14% and 19% at the first survey in N’Dakonankro, Yoho and Bozi, respectively. Only 12 subjects were found to have fever at the third survey overall. The univariate logistic regression showed that the risk to be febrile was almost halved for children who reported having slept under a net (OR = 0.54, 95% CI 0.41-0.71).

### Relationship between fever and *P. falciparum* infection

Overall the proportion of symptomatic malaria infection was 45%, 67% and 64% in N’Dakonankro, Yoho and Bozi, owing to a statistically significant difference between villages (p < 0.01, with N’Dakonankro being the lowest) but did not differ over time.

There was a poor but statistically significant association between fever and presence of *P. falciparum* infection (OR = 0.76, 95% CI 0.60-0.96). Of the total 1,482 subjects who had a recorded fever and were tested for malaria parasites by either microscopy or RDT, concordance was 0.41 and 0.43, respectively (Table 3). The predictive values of having or not having fever for malaria infection were 0.26 and 0.73.

**Table 3:** Concordance between diagnostic tests and fever among children aged <15 years in three villages of central Côte d’Ivoire

		**Fever**		
		**Negative (%)**	**Positive (%)**	**Total**
RDT	Negative (%)	388 (26.2)	147 (9.9)	535 (36.1)
	Positive (%)	698 (47.1)	249 (16.8)	947 (63.9)
	Total (%)	1,086 (73.3)	396 (26.7)	1,482 (100)
	***Accuracy***			***0.43***
Microscopy	Negative (%)	368 (24.8)	160 (10.8)	528 (35.6)
	Positive (%)	718 (48.5)	236 (15.9)	954 (64.4)
	Total (%)	1,086 (73.3)	396 (26.7)	1,482 (100)
	***Accuracy***			***0.41***

### Risk factors for *P. falciparum* infection

Table 4 summarises the results from the logistic regression analysis of *P. falciparum* infection among 1,482 persons. As to the sites, the odds of being infected were highest in Yoho (OR = 1.13, 95% CI 0.86-1.48) and lowest in Bozi (OR = 0.77, 95% CI 0.59-1.01), but there was no statistically significant difference between sites. No association was found for fever and net usage. The risk of malaria infection increased significantly with age within this cohort of children under the age of 15 years and was significantly higher during the rainy than the dry season.

**Table 4:** **Logistic regression of *P. falciparum*-positive slides in three villages, central Côte d’Ivoire**

	**Univariable model**		**Multivariable model**	
	**Odds ratio**	**95% confidence interval**	**Odds ratio**	**95% confidence interval**
N’Dakonankro	1.00		1.00	
Yoho	1.16	0.89, 1.51	1.09	0.83, 1.44
Bozi	0.82	0.63, 1.06	0.77	0.59, 1.01
Age	1.09	1.05, 1.12	1.05	1.01, 1.08
Male	1.00		1.00	
Female	0.83	0.67, 1.02	0.83	0.69, 1.04
Dry season	1.00		1.00	
Rainy season	2.56	1.97, 3.34	2.11	1.55, 2.89
No fever	1.00		1.00	
Fever	0.76	0.60, 0.96	0.95	0.74, 1.23
No net used previous night	1.00		1.00	
Nets used previous night	1.17	0.92, 1.48	1.03	0.80, 1.32

### Parasitaemia

Parasite counts were mostly low, particularly at the third survey. Of the 994 *P. falciparum*-positive slides (67.1% of the 1,482 slides examined), 314 (31.6%) had a parasite count >1,000 parasites/μl of blood. The number and proportion of children who had a positive slide with >1,000 parasites/μl of blood at the first survey was 60 (36.4%) in N’Dakonankro; 73 (40.3%) in Yoho; and 50 (36.0%) in Bozi. At the third survey, the respective numbers and percentages were 34 (31.2%) in N’Dakonankro; 20 (17.7%) in Yoho; and 23 (22.5%) in Bozi. The difference between the first and the third survey in the study villages was statistically significant in Bozi and Yoho (p < 0.05, with a higher proportion of lower parasitaemias at the third survey), but not N’Dakonankro.

### RDT performance

Among the 1,482 malaria tests subjected to both microscopy and RDT, 63.9% were RDT-positive and 64.4% were microscopy-positive, with moderate agreement between the two diagnostic approaches (kappa = 0.41, 95% CI 0.36-0.46). Considering microscopy as the diagnostic reference standard, RDT sensitivity at the first survey was 79.6% and specificity was 61.0%. At the third survey, sensitivity and specificity for RDT to diagnose malaria were 76.1% and 60.7% (Table 1). RDT performance was significantly lower (p < 0.001) with low parasite counts: 74% and 87% for parasitaemias <1,000 and ≥1,000 parasites/μl of blood, respectively (Table 5).

**Table 5:** **Concordance results between *P. falciparum *** **RDT and microscopy among children aged <15 years in three villages, central Côte d’Ivoire**

		**RDT**			
		**Negative**	**Positive**	**Total**	**% positive**
Microscopy	Negative	320	168	488	34
	Positive (parasitaemia <1,000 parasites/μl of blood)	174	506	680	74
	Positive (parasitaemia ≥1,000 parasites/μl of blood)	41	273	314	87
	Total	535	947	1,482	64
	% positive	40	82	67	

## Discussion

This study was conducted in an attempt to fill an important gap on the knowledge of the burden of malaria infection in Côte d’Ivoire, particularly the associations between children sleeping under a net (mainly ITN and LLIN) and malaria infection and all-cause fevers. Civil unrest, with important population movements [19], rendered this study a difficult one, and might have confounded some of the results.

Multivariable analysis showed that the risk of *Plasmodium* infection did not depend on the village of residency, presence of fever or recent net use. Conversely, as expected, the risk of malaria was related to rains and increased with age within the age-range of the population investigated (children aged <15 years). This observation is consistent with previous studies conducted in western Côte d’Ivoire [20] and in Zanzibar [21], where malaria was associated with age and season. In Zanzibar malaria incidence, as well as parasite density, increased with age (2–23 months). On the contrary, in Côte d’Ivoire, parasitological and clinical parameters decreased with age (children aged ≥5 years were less affected than their younger counterparts), with higher rates observed in the rainy season (208 *vs.* 171 asexual forms per μl of blood). Some malaria cases were observed at the end of the dry season, possibly because malaria transmission occurred year-round with probable low intensity during this season.

*Plasmodium falciparum* infection was found to be highly prevalent among children in the three study villages in central Côte d’Ivoire, corroborating previous findings [22,23]. While significantly higher during the rainy season (more than three-quarters of children infected), the risk persists all year-round with more than half of the children harbouring malaria parasites at the end of the long dry season, in all three villages.

Can fever be used as an indicator to assess the impact of preventive measures like net use on malaria risk? In the current study the answer is no. The relationship between malaria parasitaemia and fever has been reviewed recently [24]. In our population studied, having fever predicted only one in five malaria infections, while being afebrile predicted only three in four negative parasitological tests. Previous research by other scientists did find a relationship between parasitaemia and fever [25,26], providing the basis for a “pyrogenic threshold”, which would vary with the season [27]. For example, in Burkina Faso, 3,150 parasites/μl of blood in the high and 1,350 parasites/μl of blood in the low transmission season [28]. Whether fever can predict malaria might also depend on prevalence; one study found that fever was likely to be useful when infection prevalence was above 34-37% [29]. In our case, prevalence was high, but parasitaemias tended to be low, and probably below the pyrogenic threshold in many cases. Low parasitaemia may also account for the observed weak performance of RDT for malaria diagnosis. In particular, two findings of the third survey (rainy season) are unexpected: the high proportion of low parasitaemias (<1,000 parasites/μl of blood) and the very low proportion of children with fever (~48% of parasitaemic children were asymptomatic). However, the interpretation of our findings is difficult as we did not control for use of antimalarials and antipyretics. Asymptomatic carriers will pose a problem to intensify malaria control, as they will go undetected while continuing to sustain transmission [30].

The targeted catch-up distribution of LLINs in Bozi had no apparent effect on malaria infection or all-cause fevers, as compared to the other villages, but Bozi was the only village where parasitaemia decreased between the first and the third survey in the children seen on both occasions. Similar results were published previously in a comparable epidemiological setting in Côte d’Ivoire [22], which could be firstly explained by the fact that measurement of LLIN effect was carried out during a short period as implemented in the current study. In all three villages, study participants were less likely to sleep under an ITN or a LLIN at the end of the dry season (first survey) than in the period following the end of the short rainy season (second survey) or during the main rainy season (third survey) – probably an expression of perceived nuisance by mosquitoes, as shown elsewhere (e.g. in Kenya [31]). However, availability and use of nets did not seem to change the risk of *Plasmodium* infection or fever in relatively high exposure endemic areas. There was no obvious difference between N’Dakonankro where people used mainly ITNs and Yoho and Bozi where LLINs were the predominant net type. ITNs prove generally protective in children by reducing uncomplicated malaria incidence, malaria parasite infection prevalence and malaria-attributable mortality [2,32]. For example, ITN users in western Kenya highlands were 30% less sick from malaria than non-users in the rainy season [31], whilst in Nigeria, in hospitals, the number of malaria attacks was reduced among ITN-users [33]. A possible explanation of our findings might be that the short study time (<2 year) prevented detecting effects; various studies have reported decreases in malaria infection after 2 years [34] or 3 years [35] of follow-up in areas with high LLIN coverage.

There was only moderate agreement between RDT and microscopy; both sensitivity and specificity of RDT were lower than expected [9,36]. Sensitivity appeared to be related to parasitaemia: 87% for parasite counts ≥1,000 per μl of blood *vs.* 74% for counts <1,000 parasites/μl of blood. The overall prevalence ranged between 50% and 80%. These results could be explained by the fact that RDT based on *Pf*HRP2 detection on the one hand do not reliably detect lower-density parasitaemia [37], and on the other hand that *Pf*HRP2 persists several days after parasites disappear [38].

Our study has several limitations. First, seasonal differences between surveys might have been a confounding factor for the lack of association between net coverage and the risk of malaria. It should be noted, however, that the three villages experienced average and comparable rainfall over the course of the study. Second, we did not collect information on factors that might have impounded on the association between malaria risk and net usage, such as socioeconomic status, parents’ education attainment, household size, exact number and type of net per household and per person and number of sleeping spaces protected by nets. Third, there was substantial patient attrition between surveys (~80% and ~50 of the original cohort missing at the second and third survey, respectively). This was largely due to the sociopolitical political crisis of 2010, which pushed people to move into secured areas; when the war was over, the return of displaced populations was progressive while those who did not move were not primarily concerned with the study. However, the relatively large sample size (~300 children were seen both at the first and third survey) should at least partly overcome this limitation.

## Conclusion

Lessons learned from this study are that surveys are important to target interventions, and should cover a period sufficiently long to detect trends and quantitate the effects of interventions. The suitability of RDT in these conditions should be studied in more detail. Although our study did not show any appreciable associations between net usage and malaria infections or fevers, the use of nets (mainly LLINs) may have been associated with lower parasitaemia.

## Abbreviations

ACT: Artemisinin-based combination therapy; CHW: Community health worker; CI: Confidence interval; CSRS: Centre Suisse de Recherches Scientifiques en Côte d’Ivoire; EIR: Entomological inoculation rate; HRP2: Histidine rich protein 2; IRS: Indoor residual spraying; ITN: Insecticide-treated net; LLIN: Long-lasting insecticidal net; OR: Odds ratio; PNLP: Programme National de Lutte contre le Paludisme; RDT: Rapid diagnostic test.

## Competing interests

The authors declare that there are no competing interests.

## Authors’ contribution

AFO implemented the study, analysed the data and drafted the manuscript. PLO assisted with the data analysis and revision of the manuscript. MD, GR and MT contributed to the design of the study and the revisions of the manuscript. JU contributed to the design of the study and assisted in the drafting and revision of the manuscript. BGK designed the study, coordinated field activities and assisted with the data analysis and revision of the manuscript. All authors read and approved the final manuscript.
